# Age-Related Differences in the Physical and Physiological Demands during Small-Sided Games with Floaters

**DOI:** 10.3390/sports7040079

**Published:** 2019-04-02

**Authors:** Alberto Rábano-Muñoz, Jose Asian-Clemente, Eduardo Sáez de Villarreal, Jack Nayler, Bernardo Requena

**Affiliations:** 1 Football Science Institute, Granada 18001, Spain; albertorabmun@gmail.com (A.R.-M.); josasicle@gmail.com (J.A.-C.); bernardorequena@icloud.com (B.R.); 2 Physical Performance Sports Research Center (PPSRC), Universidad Pablo de Olavide, Sevilla 41013, Spain; 3 Department of Sport sciences, Universidad Pablo de Olavide, Sevilla 41013, Spain; 4 The Celtic Football Club Performance Department, Glasgow G11 6BZ, UK; jacknayler@hotmail.co.uk

**Keywords:** football, soccer, training, movement patterns, GPS, time-motion, youth players

## Abstract

The purpose of this study was to compare the physical and physiological demands of a small-sided game (SSG) in three different age groups (senior, under-19 [U-19] and under-17 [U-17]) belonging to the same academy. A further aim was to contrast the physical and physiological profiles of normal and floater players during this task. Thirty male football players performed a 4 vs. 4 + 2 floaters on a playing field of 40 by 30m for four bouts of 4 min with 2 min of passive recovery. In addition to heart rate (mean and maximal), a GPS (Global Positioning System) system was used to record the distances covered at different speeds, the number of accelerations and decelerations, and the work/rest ratio (W:R Ratio). Analysis of the data showed that the demands of the SSGs are determined by the age of the players and that the regular players have greater demands than floater players in the SSGs utilized. These results suggest that the coaches should pay attention to the promotion of players to superior teams because there are physical differences between them (especially the U-17 to U-19 teams). Likewise, coaches should understand that floaters are a useful tool for regulating the training load of players and programming the return-to-play process, as floater players experience lower demands than normal players.

## 1. Introduction

Small-sided games (SSGs) represent modified football games played on reduced pitch areas, often using adapted rules and involving a smaller number of players than full-size soccer matches [1]. SSGs are one of the most common drills used by coaches in their daily training to develop technical and tactical skills, as well as the endurance capacity of their players [2]. In the last few years, many research studies have focused on analyzing how the diverse constraints applied in these games affects the responses of the players [3]. The most common responses analyzed are physiological (heart rate, blood lactate), physical (distances, speeds, time), perceptual, and technical [1,3,4]. Although the most investigated variables are the pitch size, player number and the multiple combinations of them [5,6], there are other aspects that have received attention such as inclusion of goalkeepers [7,8], duration of bouts [9,10], coach encouragement [11], number of touches allowed per player and the method of defending [12,13]. In general, results suggest that the fewer the number of players that are involved, the greater the physiological and perceptual responses and the number of technical actions [1,3,14].

Nowadays, SSGs have become a useful resource to train players of all ages and competitive levels [15]. In soccer academies, it is common for players of different ages to perform similar SSGs. Surprisingly, to our knowledge, no study has documented responses to an identical SSG task in players of a similar competitive level but different age. There are studies that examine the age-related differences in the physical and physiological demands of youth players during matches [16], and age-related differences in physical capacities and their correlation with soccer-related physical performance [17]. Such studies have also demonstrated that tactical behavior of players varies during the same SSG with the age groups of under-9, under-11 and under-13 [18], and under-16, under-17 and under-19 [19]. Despite this, there is no information about the effect of age on the physical responses in the same training exercise. 

A common practice in elite-soccer academies is the movement of players between teams. In some cases, individuals are recruited by higher teams to train and compete with them due to the player’s skill in soccer and their high performance in matches. In other cases, the reduction of players in a squad as a result of injury or infraction events (e.g., players sent off) may also lead to player recruitment. Additionally, as a consequence of technical decisions or occasional occurrences, the movement of players has become standard practice during the soccer season. In particular, this practice occurs between the under-23 (senior), under-19 and under-17 age groups. Each of these teams have their specific player age ranges: Under-17 (players > 16 and ≤ 17 years old), under-19 (players > 17 and ≤ 19 years old) and senior (players > 19 years old). However, one player may train for three days with players of his age group, and then with another team for the two days before a competitive match. Understanding the weekly workload variations of the teams according to the competition and the developmental ages of the players (with more technical skills in the younger players and more intense weeks with an increase in age) [15]—and that an inadequate, excessive or rapid increase in training loads could result in increased soft-tissue injuries, reduced fitness and poor performance [20,21]—coaches should manage the physical demands of SSG tasks in different age groups to optimize player performance and prevent overuse injuries. 

A common rule used in SSGs design is the use of floater players. The floater is a special player who participates with the two teams in the offensive phase, always remaining with the team in possession of the ball [22]. With the use of floater players, practitioners aim to create an imbalance, with the intention of making the SSG drill more representative of a real game. Soccer is frequently played with a numerical imbalance, either momentarily or permanently [13]. However, most of the related literature has been focused on SSGs with the same numbers of players [23]. Only two studies, with differing results, have compared the responses of normal and floater players in SSGs. Hill-Haas et al. [24] found that the floaters travelled greater total distances and completed more sprints than normal players. Recently, Lacome et al. [22] demonstrated that locomotor activity and external mechanical load were lower in floaters compared with regular players, independent of the size (large and small) and type (possession game and game simulations) of SSGs. These authors suggested that the floater position could be administered to players for whom a lower physical demand would be beneficial (i.e., the youngest player or a recently injured player in a team). Taking this into account, it is necessary to examine if the floater players always have lower demands than normal players for different age ranges in these drills.

Therefore, the aims of the present study were to 1) compare the movement and physiological demands of the same SSG in three consecutive teams (senior, under-19 and under-17) of the same academy, and 2) contrast the physical and physiological profile of normal and floater players in this task. 

## 2. Method

### 2.1. Participants

Thirty male football players divided into three different age groups—under 17 years (U-17), under 19 years (U-19) and senior semi-professional players (SP)—participated in this study. Athletes were members of a semi-professional Spanish team, each with more than 8.5 years of experience playing soccer prior to the commencement of the study. Their standard weeks always involved four sessions in which the coaches employed SSGs for the majority of training to improve player fitness and prepare them for competition. Goalkeepers and players who had been injured during the season were excluded. This work was conducted according to the ethical standards in sport and exercise science research [25]. All the players who participated were notified about the aims of the investigation and gave their informed consent before the study began. Characteristics of the players were described in the Table 1. 

### 2.2. Procedures

Three weeks of data was collected during the 2017–2018 competitive season. Players participated in four training sessions per week (between 80 min and 120 min of duration) and one competitive match (Sunday). Measurements took place on the day after the day off, when more difficult training sessions were performed (Wednesday). All of these sessions started with the same 20 min warm-up based on mobility and active stretching, and were completed on the same artificial turf and at the same time of day (20:00–22:00 pm).

**Figure 1:**
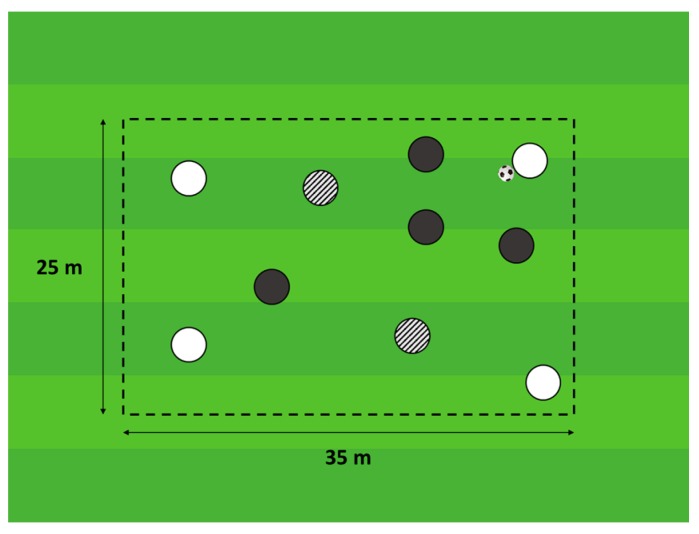
Format of the small-sided games (SSG), 4 vs. 4 small-sided games with two internal floaters.

The SSG performed in this study—4 vs. 4 + 2 floaters, as shown in Figure 1—was frequently used as a part of the training, so the participants were highly familiarized with this task. Each team in the SSGs was balanced according to technical and tactical level, competitive experience, player positions and the subjective evaluation of the coaches [5,26]. The two teams of 4 players participated in the SSG with the aim of keeping possession while they were supported by two floaters, who always assumed an offensive role to create an offensive numerical superiority [27]. The drill occurred on a pitch size of 40 m × 30 m (relative area per player = 150 m^2^), and was played for four bouts of 4 min with 2 min of passive recovery. 

The SSGs were performed without a limit on the number of ball touches and with player-to-player marking. To avoid any disruption of play, footballs were deposited around the edge of the pitch. Coaches verbally encouraged the players to maintain a high work rate during the SSG bouts. 

### 2.3. Measures

Movement performance parameters were monitored using a GPS system (GPSports SPI Elite System, Canberra, Australia) with a sampling rate of 5 Hz. These devices have previously been validated for measuring time-motion characteristics in team sports [28,29]. The distances covered at varying speeds were recorded using different thresholds as described in previous research [30]. The same approach was used for the number of accelerations and decelerations [31,32] and the work/rest ratio (W:R Ratio) [27,30]. Each player wore a heart rate monitor (Polar Team 2^®^, Polar Electro Oy, Finland) to obtain the values of maximal and mean heart rate (HR_max_ and HR_mean_, respectively). 

### 2.4. Statistical Analysis

The data presented in this study is given as a mean ± standard deviation (SD). All variables presented normal distribution (Shapiro-Wilk Test). A repeated-measures analysis of variance was used to determine differences in the distance covered in each speed zone, accelerations and decelerations, maximal velocity and meters per minute covered. Cohen’s effect size (ES) was also calculated to compare the magnitude of the differences between groups on certain variables and quantitative differences were assessed qualitatively [31] as: <1%, almost certainly not; 1−5%, very unlikely; 5−25%, unlikely; 25−75%, possible; 75−95%, probable; 95−99%, very likely; and >99%, almost certain. A substantial effect was set at >75% [7,19]. If the chance of higher or lower differences was >75%, the true difference was assessed as clear. The SPSS statistical software package (V20.0 for Windows, SPSS Inc., Chicago, IL, USA) was used for data analysis.

## 3. Results

Comparisons between the external and internal load during the SSG are arranged by age group in Table 2.

### 3.1. Comparison between Age Groups

Significant differences were found in the movement demands between the three age groups. The U-17 group presented substantially lower values with respect to U-19 and SP in total distance covered (1733.2 ± 167.6 vs. 1963.6 ± 119.7 and 1957 ± 145.5), distance covered at 7–13.9 km·h^−1^ (818.4 ± 190.3 vs. 936.5 ± 134.9 and 960.2 ± 131.8), distance covered at 14–17.9 km·h^−1^ (159.6 ± 31 vs. 293.1 ± 93.1 and 288.6 ± 81.7), distance covered at >18 km·h^−1^ (5.4 ± 3.6 vs. 20.7 ± 16.5 and 37.1 ± 23.9), W:R Ratio (6.52 ± 1.7 vs. 9.21 ± 2.57 and 9.89 ± 2.02), and number of accelerations (13.5 ± 3.6 vs. 19.8 ± 13.5 and 20.7 ± 5.1). This group only reached higher values than the U-19 and SP groups in distance covered at 0-6.9 km·h^−1^ (749.4 ± 58.6 vs. 705.5 ±72.9 and 671.1 ± 62.5, respectively). Similarly, the U-17 group presented substantially lower values with respect to U-19 in the number of decelerations (24.5 ± 7.7 vs. 38.4 ± 9.3). The U-19 group covered substantially less distance than the SP group between 7 and 13.9 km·h^−1^ (936.5 ± 134.9 vs. 960.2 ± 131.8) and distance covered at >18 km·h^−1^ (20.7 ± 16.5 vs. 37.1 ± 23.9). Nevertheless, the U-19 group presents a higher number of decelerations (38.4 ± 9.3 vs. 26.7 ± 8.6) and a higher HR_mean_ (170.6 ± 13.5 vs. 160.4 ± 9.7) when compared to the SP group. The heart rate demands of the U-17 group also presented lower values than U-19 and SP in HR_mean_ (155.5 ± 17.7 vs. 170.6 ± 13.5 and 160.4 ± 9.7, respectively).

### 3.2. Comparison between Regular and Floater Players

Floater players presented lower values in most of the variables analyzed for all age groups when compared to regular players, as shown in Table 2. In the SP group, floater players demonstrated lower values than regular players in total distance (1508.8 ± 160 vs. 1957.0 ± 145.5), distance covered at 7–13.9 km·h^−1^ (616.6 ± 74.1 vs. 960.2 ± 131.8), distance covered at 14-17.9 km·h^−1^ (144.9 ± 36.4 vs. 288.6 ± 81.7), distance covered at >18 km·h^−1^ (7.0 ± 3.5 vs. 37.1 ± 23,9), W:R Ratio (4.74 ± 0.96 vs. 9.89 ± 2.02), number of accelerations (12.0 ± 2.65 vs. 20.7 ± 5.1), number of decelerations (13.0 ± 2.7 vs. 26.7 ± 8.6) and HR_mean_ (152.5 ± 21.7 vs. 160.4 ± 9.7). For the U-19 group, similar results were obtained and floater players presented inferior values in total distance (1725.8 ± 223.3 vs. 1963.6 ± 119.7), distance covered at 7-13.9 km·h^−1^ (751.3 ± 294.2 vs. 936.5 ± 134.9), distance covered at 14–17.9 km·h^−1^ (201.2 ± 16.7 vs. 293.1 ± 93.1), distance covered at >18 km·h^−1^ (14.6 ± 1.2 vs. 20.7 ± 16.5), W:R Ratio (6.75 ± 2.49 vs. 9.21 ± 2.57), number of accelerations (13.5 ± 2.1 vs. 19.8 ± 7.9), number of decelerations (23.0 ± 2.8 vs. 38.4 ± 9.3) and HR_mean_ (155.8 ± 17.0 vs. 170.6 ± 13.5). These results were also consistent with the findings for the U-17 group, with the floater players obtaining smaller values for total distance (1531.7 ± 116.7 vs. 1733.2 ± 167.6), distance covered at 7-13.9 km·h^−1^ (643.6 ± 100.1 vs. 818.4 ± 190.3), distance covered at 14–17.9 km·h^−1^ (38.7 ± 13.5 vs. 159.6 ± 31.0), W:R Ratio (3.2 ± 2.8 vs. 6.52 ± 1.7), number of accelerations (9.7 ± 2.4 vs. 13.5 ± 3.6), number of decelerations (15.6 ± 0.6 vs. 24.5 ± 7.7) and HR_mean_ (132.6 ± 25.6 vs. 155.5 ± 17.7).

## 4. Discussion

The main aim of this study was to analyze the physical and physiological demands during a frequently used SSG with floaters (4 vs. 4 + 2) in three different age groups of elite players belonging to the same academy. Further, an analysis of the performance of regular and floater players was performed. The main findings demonstrated that 1) the demands of the drills are determined by the age of the players and 2) regular players have greater demands than floaters players in the SSG utilized.

There is no previous research that compares age related differences between the same SSGs, however analysis of match demands in elite youth football indicates that total distance covered increases with age [16]. The results of the present research are in line with this study, showing that all groups have different movement demands. The groups SP and U-19 covered a higher total distance and relative distance than U-17. This study is also the first to compare SP and U-19 players, and the results indicate the potential existence of a ceiling effect from 19 years of age in terms of total distance covered. With regard to high-speed efforts, our findings are consistent with the literature [16], showing that >18 km·h^−1^ activity is influenced by age and an increase in age is accompanied by greater levels of high velocity movement in soccer activities. In accordance with our results, a previous study found differences in the acceleration, maximum running speed and repeated sprint performance in highly trained young male soccer players for under-14, under-16 and under-19 groups [17]. The variability in performance could be attributed to differences in the biological maturation, which allow older players to better prepare for, and therefore achieve, efforts of high intensity. Our findings could also be explained using a technical–tactical approach. The literature indicates that soccer players belonging to a higher playing division cover more high-speed running and sprint distances in the task than players of a lower level [25]. Thus, in the present study, the experience and ability of older players could account for the greater accumulated effort and velocity values observed when compared to their younger colleagues.

Focusing on maximal speed performance, there were no significant differences between groups. This is probably due to the limitation of SSGs in producing high-speed activities [30], because they are played on smaller pitch areas [32] where the players do not have enough space to reach their maximal sprinting speed. For this reason, and until future research is able to implement SSGs with a special focus on the development of high speed movement, acceleration and deceleration profiles could provide useful information about high intensity actions [33]. Concerning the number of accelerations and decelerations, the SP and U-19 groups reached a greater number of accelerations and decelerations than the U-17 group. This finding is relevant considering accelerations and decelerations are an important part of the neuromuscular load in football-specific training [33].

Physiological responses showed a different behavior depending on the variable analyzed. While data of HR_max_ was similar in the three age groups, HR_mean_ exhibited distinct values for SP, U-19 and U-17 group. Taking into account the fact that the U-19 group had a greater HR_mean_ than the SP group despite having had similar physical demands, it may be posited that the SP group possessed better fitness levels—an assertion which has previously been suggested in the literature [26]. In spite of the differences the HR values showed, the SSGs studied could be an adequate stimulus for aerobic training of these age groups because the reported values were close to the 80–85% of HR_max_ for the players in the majority of cases [34,35].

The use of floater players is a normal practice for coaches to replicate specific game situations [36], although, to our knowledge, only two studies have compared responses of regular and floater players during SSGs [29,34], and they found contradictory results. In our study, the floater players had lower physical and physiological demands than normal players in all of the variables analyzed except the highest speed reached and HR_max_, where there were no differences between the two groups. Thus, the results of this study concur with the perspective presented by Lacome et al., [22] wherein the floater players experience a lower load than normal players. These findings support the concept that floaters can be used by coaches to minimize the training load in some special events (players overtraining, after injury or when they are recruited to train with an older team), allowing such players to train with teammates while receiving a specific and particular load.

One of the limitations of this study was its scope, in that only three age groups from the superior academy and only one task type was evaluated. As a result, future work could focus on comparing all the age categories and researching a range of common drills used in soccer.

## 5. Conclusions

The data of the present study demonstrated that during a frequent SSG of football employing a 4 vs. 4 + 2 floaters format, the load received by the soccer players and the role of normal or floater players during the task was different for each age group:

- The demands of the drills were determined by the age of the players, showing greater performance with increasing age. Particular attention should be given to the promotion of U-17 players to U-19 teams for development, as substantial differences in physical behavior exist between the two age groups. Coaches should take into account these observations, and be aware of such differences in order to prioritize the technical–tactical talent of the players (especially with respect to younger players), thereby avoiding possible ageism. In a soccer academy, not only players with greater physical performances should be promoted, but also players with lesser physical performances, particularly those with greater abilities and skills to play soccer.
- Regular players had greater demands than floater players in the SSG utilized, so technical staff aiming to minimize the load of particular players may practically apply the floater role. It is generally accepted by the soccer community that players that have overtrained or have injuries may be employed as floaters to minimize training stress. From this perspective, our data offers a new and interesting approach in which floaters could be used to reduce the physical impact of players promoted in lower age teams.
- Understanding the physical and physiological demands of a task in soccer, as well as all the possible options used for increasing or decreasing the load (e.g., floaters). The use of floaters should be one of the main concerns of coaches and assistants in order to create adequate sessions and training weeks that optimize the performance and fitness of the players. This will improve the players’ preparation for matches, and in particular the most demanding phases of them [37].

## Figures and Tables

**Table 1:** Participant characteristics.

Group	Age (years)	Height (cm)	Weight (kg)	Experience (years)
SP	24.09 ± 3.51	177.18 ± 5.91	70.27 ± 8.19	13.18 ± 2.96
U-19	17.73 ± 0.85	175.1 ± 6.42	65.67 ± 7.39	8.64 ± 1.86
U-17	15.97 ± 0.58	171.2 ± 5.57	60.49 ± 5.95	6.45 ± 1.61

Note: The data represents means and standard deviations, with SP = Senior players; U-19 = Under 19 players and U-17 = Under 17 players.

**Table 2:** Physical and physiological demands of the normal and floater players during 4 vs. 4 + 2 floaters SSG.

*Variable*	SP		U-19		U-17	
	Regular	Floater	Regular	Floater	Regular	Floater
Total Distance	1957.0 ± 145.5	1508.8 ± 160.0 *	1963.6 ± 119.7	1725.8 ± 223.3 *	1733.2 ± 167.6 ^s, j^	1531.7 ± 116.7 *
Maximal Speed	20.7 ± 1.2	20.3 ± 1.4 *	20.6 ± 1.2	21.15 ± 4.7	19.4 ± 1.2	18.5 ± 3.1
Distance 0–6.9 km·h^−1^	671.1 ± 62.5 ^c^	740.3 ± 106.0	705.5 ± 72.9 ^c^	758.4 ± 72.9	749.4 ± 58.6	846.7 ± 34.8
Distance 7–13.9 km·h^−1^	960.2 ± 131.8	616.6 ± 74.1*	936.5 ± 134.9 ^s^	751.3 ± 294.2 *	818.4 ± 190.3 ^s, j^	643.6 ± 100.1*
Distance 14–17.9 km·h^−1^	288.6 ± 81.7	144.9 ± 36.4 *	293.1 ± 93.1	201.2 ± 16.7 *	159.6 ± 31.0 ^s, j^	38.7 ± 13.5 *
Distance >18 km·h^−1^	37.1 ± 23.9	7.0 ± 3.5 *	20.7 ± 16.5 ^s^	14.6 ± 1.2 *	5.4 ± 3.6 ^s, j^	3.2 ± 2.8
W:R Ratio	9.89 ± 2.02	4.74 ± 0.96 *	9.21 ± 2.57	6.75 ± 2.49 *	6.52 ± 1.7 ^s, j^	4.56 ± 0.6 *
Acc > 2.5 m·s^−2^	20.7 ± 5.1	12.0 ± 2.65 *	19.8 ± 7.9	13.5 ± 2.1 *	13.5 ± 3.6 ^s, j^	9.7 ± 2.4 *
Dec > 2.5 m·s^−2^	26.7 ± 8.6 ^j^	13.0 ± 2.7 *	38.4 ± 9.3	23.0 ± 2.8 *	24.5 ± 7.7 ^j^	15.6 ± 0.6 *
HR_mean_	160.4 ± 9.7 ^j^	152.5 ± 21.7 *	170.6 ± 13.5	155.8 ± 17.0 *	155.5 ± 17.7 ^s, j^	132.6 ± 25.6 *
HR_max_	183.6 ± 6.8	179.7 ± 15.1	188.9 ± 12.8	186.5 ± 2.1	185.6 ± 8.9	184.5 ± 9.2

Note: The data represents means and standard deviations, with s indicating substantial differences with respect to SP, j indicating substantial differences with respect to U-19, and c indicating substantial differences with respect to U-17. The * designates that substantial differences between floater and normal players were present.
